# The Association of Autoantibodies in Hyperthyroid Heart Disease Combined with Pulmonary Hypertension

**DOI:** 10.1155/2019/9325289

**Published:** 2019-12-01

**Authors:** Xiujuan Zhang, Lin Chen, Jianping Sheng, Chaoying Li, Yong He, WenXia Han

**Affiliations:** ^1^Department of Endocrinology, Shandong Provincial Hospital Affiliated to Shandong University, Shandong First Medical University & Shandong Academy of Medical Sciences, Jinan, China; ^2^Department of Breast and Thyroid Surgery, Shandong Provincial Hospital Affiliated to Shandong University, Shandong University Cheeloo College of Medicine, Jinan, Shandong, China; ^3^Department of Medicine, The People Hospital of Huaiyin Jinan, Jinan, Shandong, China; ^4^Department of Endocrinology, Zaozhuang Municipal Hospital, Jinan, Shandong, China; ^5^School of Management, Shandong University of Traditional Chinese Medicine, Jinan, China

## Abstract

Hyperthyroidism is a clinical state that results from increased thyroid hormone levels which has a significant impact on cardiac function and structure. Graves' disease is the most common cause of hyperthyroidism in iodine-replete areas. Hyperthyroid heart disease may be associated with pulmonary hypertension in patients who have overt hyperthyroidism. To investigate the association of pulmonary hypertension induced by hyperthyroid heart disease and autoantibody, one hundred and one cases with hyperthyroid heart disease who were consecutively admitted to the inpatient department of endocrinology and metabolism of the Shandong Provincial Hospital between November 2014 and April 2018 were collected and analyzed statistically. According to the Independent samples *T*-test, variance analysis, chi-square test, Pearson linear correlation analysis, and logistic regression, there was a good correlation between pulmonary artery systolic pressure and thyroid stimulating hormone (TSH) and receptor antibodies (TRAb) (*r* = 0.264, *P*=0.025) (OR = 1.037, *P*=0.029), but there was no significant correlation between the pulmonary artery systolic pressure and other thyroid-related parameters (FT3, FT4, TSH, anti-TPO, and anti-TG). Based on variance analysis, PASP rose as the level of TRAb gets higher. What is more, patients with HHD combined with PH showed a significantly higher serum level of TRAb; moreover, serum TRAb concentration was remarkably correlated with the PASP level. Therefore, TRAb participates in the process of pulmonary hypertension caused by hyperthyroid heart disease.

## 1. Introduction

Graves' disease (GD) is one of the most common endocrine diseases with prominent cardiovascular manifestations, which is responsible for 80% of hyperthyroidism in areas of iodine sufficiency. The population is generally susceptible, especially prevalent in women aged 40–60 years [1].

The pathogenesis of GD is complex since both genetic and environmental factors can result in the attack of GD among susceptible populations, but thyrotropin receptor antibody (TRAb) ultimately accounts for hyperthyroidism [2]. The bond between thyroid stimulating antibody (TSAb) and thyrotropin receptors on the surface of thyroid follicular cells could lead to unbridled and sustained thyroid stimulation, and this kind of combination aforementioned is associated with the massive synthesis and release of thyroid hormones.

It has been reported that excessive secretion of thyroid hormones could give rise to deleterious clinical consequences for the cardiovascular system. Except for thyroid gland, susceptive receptors exist in both myocardial and vascular endothelial tissues, enabling even slight changes in circulating thyroid hormones to generate infaust influence on multiple organs and systems. Abnormal combination of receptors in the cardiovascular system could create a hyperdynamic state [3], which is characterized by increased heart rate, blood volume, and left ventricular contractility and decreased systemic vascular resistance [4]. TRAb have long been used as an indicator to evaluate the prognosis of GD and discontinuation of antithyroid drugs (ATD) [5]. Also, the patients who display elevated TRAb levels at onset are more likely to recur in the short term [6].

Positive rates of other thyroid autoantibodies, such as anti-thyroid peroxidase antibody (TPOAb) and anti-thyroglobulin antibody (TgAb), are also significantly increased in Graves' disease patients, which demonstrate that Graves' disease is an autoimmune thyroid disease from another point of view.

For the past few decades, mounting evidences have demonstrated that hyperthyroid heart disease (HHD) may be associated with pulmonary hypertension (PH) in patients who have overt hyperthyroidism. According to several observational studies, the prevalence of pulmonary hypertension in patients with hyperthyroidism varies between 35 and 47% [7–9].

Pulmonary hypertension (PH) is an infrequent, progressive, and fatal disease characterized by abnormally sustained elevations in pulmonary artery pressure and progressive increase in pulmonary vascular resistance. In most cases, due to the lack of early-stage clinical manifestations, the patients with pulmonary hypertension once exhibit obvious clinical symptoms and signs, dyspnea on exertion (DOE), for instance, has already developed severe decline in right ventricular function, and this is one of the primary causes of hospitalization and death.

Therefore, as for patients with HHD complicated with PH, close monitoring and early intervention of pulmonary arterial systolic pressure (PASP) together with thyroid function can prevent the deterioration of hemodynamics as well as the occurrence and development of heart failure [10].

Previous studies have shown that autoimmunity plays a significant role in the pathogenesis of thyroid disorder; meanwhile, the increase of cardiac output is an essential cause of hyperthyroid heart disease with pulmonary hypertension. Nevertheless, to our knowledge, no study to date has examined the contribution of thyroid hormones and autoantibodies to the development of pulmonary hypertension among hyperthyroidism patients with cardiovascular complications. Hence, the aim of this study was to evaluate thyroid autoimmunity and thyroid function in patients suffering from hyperthyroid heart disease complicated with pulmonary hypertension.

## 2. Materials and Methods

### 2.1. Patients

One hundred and one cases with hyperthyroid heart disease who were consecutively admitted to the inpatient department of endocrinology and metabolism of the Shandong Provincial Hospital between November 2014 and April 2018 were included in the study. Untreated Graves' disease patients at onset were diagnosed with HHD by at least two experienced physicians, on the basis of common clinical and laboratory criteria.

### 2.2. Diagnostic and Inclusion Criteria

#### 2.2.1. Diagnostic Criteria for Hyperthyroid Heart Disease [11, 12]

1. Confirmed hyperthyroidism case according to medical history, clinical symptoms and signs, and test results
2. Patients were included if they had one or more of the following: arrhythmia such as paroxysmal or persistent atrial fibrillation, frequent ventricular premature beat, conduction block, paroxysmal supraventricular tachycardia; cardiac dilatation; congestive heart failure; angina pectoris or myocardial infarction; and valve prolapse with pathological murmur in the cardiac auscultation area
3. The above abnormalities disappeared or improved significantly after clinical cure or complete remission of hyperthyroidism
4. Excluded other causes of heart disease
5. All patients were diagnosed with hyperthyroidism and hyperthyroid heart disease for the first time, and they did not receive any kind of treatment

#### 2.2.2. Diagnostic Criteria for Pulmonary Hypertension [13]

Pulmonary hypertension (PH) was defined by a mean pulmonary arterial systolic pressure ≥25 mmHg at rest.

#### 2.2.3. Inclusion Criteria

Inclusion criteria include the following: (1) patients who met the diagnostic criteria for HHD were enrolled in the study and (2) patients with complete data.

#### 2.2.4. Exclusion Criteria

Exclusion criteria include (1) patients with a medical history of thyroidectomy and (2) patients with a history of untreated hypertension or any disease which would affect the heart condition.

### 2.3. Methods

#### 2.3.1. Routine Examination

For each patient, the following tests and examinations were undertaken: detailed physical examination, blood pressure, heart rate, pulse, weight, routine blood count, routine urine test, liver and kidney functions, electrocardiogram, and chest X-ray films (PA position).

#### 2.3.2. Calculation of Pulmonary Artery Systolic Pressure

Transthoracic echocardiographic evaluations were performed by experienced technicians, who were blinded to the clinical findings, with a Vivid 7 echocardiographic ultrasound device (GE Vingmed Ultrasound AS, Horten, Norway) to measure the peak flow velocity of the tricuspid valve which was used in the calculation of the pulmonary artery systolic pressure using the modified Bernoulli equation: (PASP)=4 × *V*_max_^2^+10 mmHg.

#### 2.3.3. Measurement of Thyroid Function

All measurements were performed using the chemiluminescent immunometric assay (Roche Diagnostics GmbH 2010). The interassay coefficients of variation (CVs) for FT3, FT4, and TSH were 4.3%, 4.6%, and 4.8%. Interassay CVs for FT3, FT4, and TSH were 6.1%, 6.4%, and 6.7%. The patients have no history of iodine exposure, and no drugs affecting the hypothalamic-pituitary-thyroid axis were taken prior to blood collection [14–17].

### 2.4. Statistics

For quantitative variables, data were presented as mean ± standard deviation (SD). The independent samples *T*-test and chi-square test were used for comparisons of means between two groups of HHD patients. To investigate the contribution of thyroid hormones and autoantibodies to the development of PH among HHD patients, logistic regression analyses were performed for all subjects. Pearson linear correlation was used to evaluate the relationship with various tested markers with PASP levels among HHD patients with PH. Variance analyses were adopted to analyze whether different levels of TRAb have a significant effect on PASP. All analyses were calculated by Statistical Product and Service Solutions 22.0 (SPSS 22.0). *P* value less than 0.05 was considered as statistically significant.

## 3. Results

A total of 101 patients (38 male vs 63 female) were enrolled in the final analysis based on the inclusion and exclusion criteria. Average age was 46.9 ± 13.5 years, with age ranging from 17 to 74. Pulmonary hypertension, defined as PASP ≥ 25 mmHg, was present in 72 patients with HHD, accounting for 71.3%. All the cases tested positive for at least two kinds of antibodies, 100 cases tested positive for TRAb, 93 cases tested positive for TPOAb, and 65 cases tested positive for TgAb.

The clinical and biochemical characteristics of the patients with HHD are shown in Table 1; as illustrated, there was no statistical difference in age, gender, and serum FT3, FT4, TSH, anti-TPO, and anti-TG between the 2 groups. Nevertheless, the patients suffering from HHD combined with PH had a significantly higher serum level of TRAb compared with HHD patients exhibiting normal PASP (*P*=0.012). Out of the total 101 patients, 13 patients had the case history of hypertension (12.87%); however, all of them took drugs for hypertension by prescription and had their blood pressure under control. 71 patients had varying degrees of goiters. 22 male patients had either a current or former cigarette smoking history of at least 10 pack-years. 30 patients exhibited the symptom of atrial fibrillation.

We also found that serum TRAb levels remained independently associated with pulmonary hypertension among patients suffering from HHD (OR [95% CI], 1.037 [1.006–1.092]; *P*=0.026; after adjusting for age, FT3, FT4, and TSH), as illustrated in Table 2. That is to say, TRAb is a risk factor for HHD complicated with PH. But the relation between pulmonary hypertension and other laboratory parameters indicating thyroid function and autoimmunity did not reach statistical significance.

**Figure 1:**
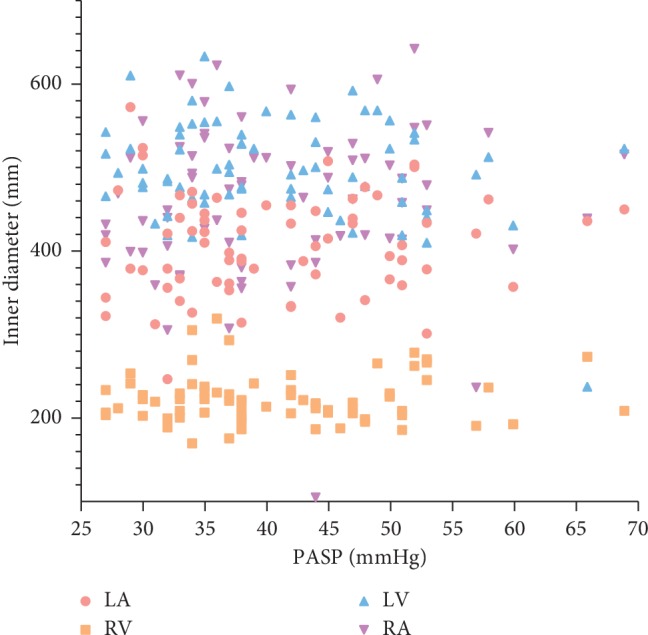
Scatter diagram of atrial and ventricular diameters of HHD patients complicated with PH.

Of all the patients, PASP was calculated by echocardiographic evaluations among 72 patients; PASP of the other 29 patients was not evaluated due to the absence of tricuspid regurgitation, but the ultrasound showed absolutely no signs of pulmonary hypertension. The echocardiographic parameters of patients suffering from HHD complicated with PH are listed in Table 3. As shown in Figure 1 and Table 3, in a total of 72 patients, 71 and 54 patients showed above normal left atrial inner diameter and right atrial inner diameter, respectively. All 72 patients exhibited varying degrees of tricuspid regurgitation, consisting in elevated maximum velocity of tricuspid blood flow and increased maximum pressure on both sides of the tricuspid valve at echocardiography.

**Figure 2:**
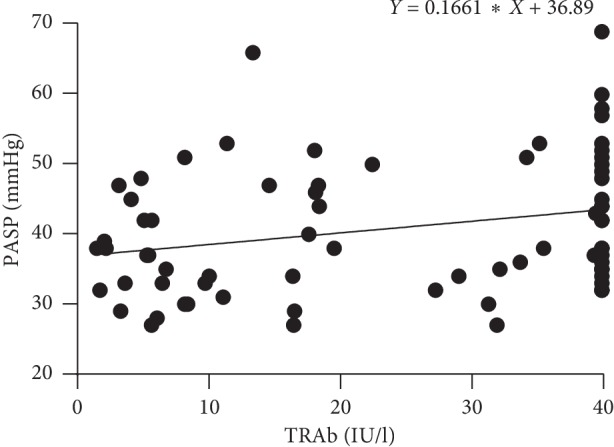
Scatter diagram of pulmonary artery systolic pressure and TRAb.

Correlation analyses of pulmonary arterial systolic pressure (PASP) with laboratory parameters were summarized in Table 4. There was a positive correlation (*P*=0.025, *r* = 0.264) between TRAb with HHD complicated with PH, as shown in the scatter diagram of PASP and serum TRAb levels (Figure 2). However, no statistically significant correlation was found between FT3, FT4, TSH, anti-TPO, anti-TG, and PASP levels (*P* > 0.05). In the next place, PASP positively correlated with a maximum velocity of blood flow in the artery of the tricuspid valve (*P* ≤ 0.001, *r* = 0.744) and maximum pressure on both sides of the tricuspid valve (*P* ≤ 0.001, *r* = 0.760), respectively.

Due to the absence of the exact number of pulmonary arterial systolic pressure in hyperthyroid heart disease patients without pulmonary hypertension, correlation analyses together with variance analysis were done between the patients suffering from HHD combined with PH instead of all subjects. We arbitrarily divided patients suffering from HHD combined with PH into 3 groups according to tertiles of the serum TRAb level (*Q*1: <11.596, *Q*2:11.596–40.000, and *Q*3: ≥40.000 IU/l), and then the levels of PASP were compared by using variance analysis; the results are presented in Table 5.

**Figure 3:**
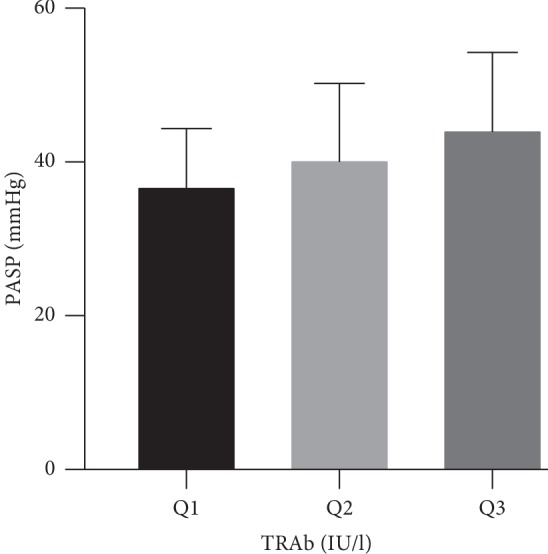
Variance analysis of serum TRAb concentration and PASP.

The comparisons of data obtained from patients who were divided into 3 groups according to the serum TRAb content are shown in Figure 3. *Q*3 was found to have significantly higher mean levels of PASP (37.1 ± 7.6 vs. 39.5 ± 8.3 vs. 44.5 ± 10.1); that is to say, it can be confirmed that PASP rises as the level of TRAb gets higher. In addition, TPOAb, maximum velocity of blood flow in the artery of tricuspid valve, and maximum pressure on both sides of the tricuspid valve increase as the level of TRAb goes up.

## 4. Discussion

While thyroid autoimmunity has been reported to be associated with thyroid hormone disorders as well as cardiovascular complications, to the best of our knowledge, this is the first retrospective study conducted investigating the association between autoantibodies and HHD complicated with PH. This study demonstrated that patients with HHD combined with PH showed a significantly higher serum level of TRAb; moreover, serum TRAb concentration was remarkably correlated with the PASP level.

Abnormal increase of circulating thyroid hormone has a close relationship with target organ damage of hyperthyroidism, for instance, the undesirable cardiovascular complications. Elevated levels of thyroid hormone can directly act on myocardium and exert positive chronotropic and inotropic effect by enhancing cardiac excitability and myocardial contraction. In addition to direct effect, high concentrations of thyroid hormone could also stimulate angiogenesis; moreover, it accelerates vascular smooth muscle cell relaxation.

Hyperthyroidism heart disease is a kind of cardiomyopathy due to metabolic disorder, which turns out to be the consequence of long-term invalid control or exacerbation of hyperthyroidism, characterized by increased pulmonary vascular resistance. Pulmonary hypertension is a hemodynamic and pathophysiological condition in which pulmonary artery pressure rises and eventually exceeds a certain threshold and may ultimately lead to right heart failure even death if without proper treatment [18]. Multiple studies have confirmed a certain correlation between pulmonary hypertension and thyroid diseases, which has attracted extensive attention from clinicians [19, 20].

These results aforementioned suggested a significant correlation between TRAb and the pathogenesis of pulmonary hypertension among HHD patients. Furthermore, the rate of HHD combined with PH patients who were tested positive for at least two types of autoantibodies reached up to 100% in our research. In this regard, Chu et al. [20] have conducted more detailed studies, suggesting an autoimmune pathogenic relationship between pulmonary hypertension and thyroid disease. Nevertheless, Park [21] revealed in their study that the substantial prevalence of patients with hyperthyroidism exhibits elevated PASP; in the meantime, their research did not support Chu et al.'s discovery.

Comparatively speaking, the positive rate of TPOAb and TGAb in the two studies aforementioned was only 54% and 35%, respectively. By contrast, we found a higher occurrence of autoimmune hyperthyroidism (99% for TRAb and 100% for at least two types of thyroid autoantibodies). In addition, there was no significant correlation between PASP and FT3, FT4, TSH, anti-TPO, and anti-TG in the HHD cases we included. However, TRAb strongly correlated with PASP. Therefore, it can be demonstrated that the elevation of pulmonary arterial systolic pressure is related with the increasing level of serum TRAb.

Although no association of FT4 with HHD complicated PH was observed in our study, a few recent retrospective cohort studies have arrived at different conclusions. Okosieme et al. observed increased risk of mortality and major adverse cardiovascular events with increasing concentrations of FT4 in serial measurements, but not for increasing concentrations of TSH. Moreover, risks of mortality and major adverse cardiovascular events were higher in older patients than in younger patients [22]. By contrast, Lillevang-Johansen et al. reported that the difference of mortality rate in treated and untreated hyperthyroidism is related to cumulative periods of low serum TSH [23].

On the contrary, our results showed that all the patients suffering from HHD complicated with PH developed various degrees of tricuspid regurgitation. These findings were consistent with what was known about the pathogenesis of right heart failure [24–30]. Tricuspid regurgitation caused by chronic pulmonary hypertension could result in the regurgitation of blood from the right ventricle through the incomplete tricuspid valve back into the right atrium. As the pressure load continuously elevated, compensatory mechanisms such as myocardial remodelling came into play. Followed by increasingly serious myocardial hypertrophy and ventricular dilatation, cardiac function deteriorated step by step. Lack of energy supply and impaired utilization of cardiomyocytes led to necrosis and fibrosis of myocardial cells; when taken together, myocardial contractility and compliance were significantly reduced. When decompensation of ventricle occurred, cardiac output weakened progressively, resulting in organ and tissue perfusion insufficiency. Although the exact mechanisms of right heart failure secondary to pulmonary hypertension remain to be further investigated, the function and size of the right ventricle plays an important role in survival time [31].

Graves' disease is an autoimmune thyroid disorder mediated by TRAb, leading to superabundant synthesis and secretion of T3 and T4, which have been verified as the main cause of hyperthyroidism. Consequently, all patients with hyperthyroidism are supposed to reexamine the TRAb level during monthly or quarterly regular follow-up. The persistent elevation of TRAb indicates that hyperthyroidism is in the active stage, while the remission phase is normally accompanied by the decline of TRAb [32, 33]. Roberto et al. found that patients who experienced recurrence(s) had 1.7-fold higher levels of TRAb at onset. Furthermore, they were more likely to show thyroid hypervascularization [6].

Nicolls et al. [34] revealed the underlying mechanism of autoimmunity in the occurrence and development of pulmonary hypertension. Abnormal combination of vascular endothelial cells and pathogenic autoantibodies could set off cell apoptosis; whereafter, pulmonary hypertension and vascular remodelling may start to take place. However, the exact pathophysiological mechanism of HHD complicated with PH has not been fully understood. It may occur as overlapping consequences of the following several theories: (1) hemodynamic changes [27]: compared with systemic circulation, pulmonary circulation presents a lower compensatory ability for hyperdynamic blood flow. Therefore, when hyperthyroidism occurs, apparent increase in cardiac output would result in PASP elevation [35]. (2) Autoimmunity could induce and aggravate pulmonary hypertension through two kinds of mechanisms: disrupt the balance between endothelium-derived relaxing factors (EDRFs) and endothelium-derived contracting factors (EDCFs) and act as a mediator to regulate the diastolic activity of vascular smooth muscles [36, 37]. (3) Sustained hypoxic pulmonary vasoconstriction (HPV), induced by the elevation of angiotensin II and angiotensin converting enzyme, contributes to the development of pulmonary hypertension and right heart failure [38, 39]. (4) The impairment of left ventricular function, caused by hyperthyroidism, would affect pulmonary hemodynamics and thus exacerbate pulmonary hypertension. (5) Inadequate adrenomedullin (ADM) [38], a vasodilatory peptide, regulates the cardiovascular system in the following ways: interferes with the dilation of blood vessels, inhibits the proliferation and migration of vascular smooth muscle cells, and reduces blood pressure. Therefore, when our body lacks sufficient compensatory effect for ADM deficiency, the resistance of pulmonary circulation increases, which might be one of the important aetiological factors for pulmonary hypertension. (6) Urotensin II is able to directly act on receptors, thereby bringing about the contraction and proliferation of vessel smooth muscle cells, all of which cause reduction in pulmonary vascular compliance and increase in vascular resistance and eventually pulmonary hypertension [32, 40].

Beyond that, multiple research studies have highlighted the value of physical or emotional stress as a trigger of Graves' disease-related hyperthyroidism. It is commonly believed that the deterioration or relapse of Graves' disease is preceded by at least one stressful event. In addition, the more stressed these patients are, the shorter is the interval between stressful events and Graves' disease hyperthyroidism. Above all, the interplay between environmental factors and susceptibility genes eventually leads to disruption of the immune tolerance [41–43].

It has been repeatedly reported that, as long as under standard treatment of hyperthyroidism, a great mass of patients developing HHD accompanied by PH would exhibit different degrees of reduction in PASP after the thyroid function returns to normal [44, 45].

In summary, our findings indicated that TRAb is closely associated with HHD combining with PH, all of which is obvious interrelated to hemodynamic alterations and myocardial damage attributed to changes in the serum thyroid hormone level. At the same time, there might be common immune and genetic mechanisms between them, which needs further investigation. The screening of pulmonary arterial pressure in patients with hyperthyroid heart disease may be able to boost the detection rate of patients with pulmonary hypertension. Furthermore, it is necessary for us to apply the research to the epidemiology and pathogenesis of pulmonary hypertension among hyperthyroidism patients, in order to improve the clinical scientific research level in this field.

There are a few limitations of our study: (1) although ultrasound showed no sign of increased PASP, the lack of accurate data of pulmonary arterial systolic pressure among hyperthyroid heart disease patients without pulmonary hypertension is the main limitation of our study. On the contrary, invasive ways such as the Swan–Ganz catheter may lead to a small risk of serious morbidity. Consequently, we used cardiac ultrasound to diagnose PH in the present study; (2) on the one hand, we only enrolled Han nationality individuals in our study, and on the other hand, the cohort of subjects was relatively small. Therefore, our conclusion may not apply to other populations.

All in all, except for the effect of thyroid hormone as well as autoantibodies on the cardiovascular system, autoimmune-mediated pulmonary arterial systolic pressure elevation may have a vital function in hyperthyroid heart disorder-linked pulmonary hypertension.

## Figures and Tables

**Table 1:** Clinical and biochemical characteristics of the patients (means ± SD).

		HHD combined with PH		Total	*P* value
		Present (*n* = 72)	Absent (*n* = 29)		
Sex (M : F)		28 : 44	10 : 19	38 : 63	0.679
Smoking status (P : A)		16 : 56	6 : 23	22 : 79	0.866
Goiter (P : A)		52 : 20	19 : 10	71 : 30	0.505
I		8 : 64	4 : 25	12 : 89	
II		32 : 40	8 : 21	40 : 61	
III		12 : 60	7 : 22	19 : 82	
Hypertension (P : A)		9 : 63	4 : 25	13 : 88	0.861
Atrial fibrillation (P : A)		24 : 48	7 : 22	31 : 70	0.365
Systolic BP (mmHg)		126.4 ± 16.0	126.9 ± 14.5	126.6 ± 15.5	0.880
Diastolic BP (mmHg)		76.8 ± 11.3	79.2 ± 10.2	77.5 ± 11.0	0.327
BMI		22.3 ± 2.9	22.9 ± 3.9	22.5 ± 3.2	0.326
Age (years)		47.5 ± 13.4	45.7 ± 13.9	46.9 ± 13.5	0.564
FT3 (pmol/l)		22.3 ± 11.2	20.5 ± 10.9	21.8 ± 11.1	0.447
FT4 (pmol/l)		67.6 ± 35.6	64.5 ± 42.2	66.8 ± 37.4	0.695
TSH (mIU/l)		0.0075 ± 0.0099	1.713 ± 9.019	0.497 ± 4.835	0.317
TRAb (IU/l)		23.9 ± 15.1	16.9 ± 11.0	21.9 ± 14.4	0.012
Anti-TPO (IU/ml)		852.3 ± 503.8	767.4 ± 538.1	827.9 ± 512.6	0.454
Anti-TG (IU/ml)		329.5 ± 511.1	366.5 ± 528.9	333.7 ± 514.1	0.687

TSH, thyroid stimulating hormone; FT4, free tetraiodotronin; FT3, free triiodotronin; TPOAb, anti-thyroid peroxidase antibody; TGAb, anti-thyroglobulin antibodies; TRAb, thyrotropin receptor antibody; BMI, body mass index.

**Table 2:** Univariate logistic regression analysis of factors associated with HHD complicated with PH.

Variables	Univariate analysis			Multivariate analysis		
	OR	95% CI	*P* value	OR	95% CI	*P* value
Age (years)	1.010	0.978–1.042	0.560	1.020	0.984–1.057	0.280
FT3 (pmol/l)	1.016	0.976–1.057	0.443	0.989	0.926–1.057	0.753
FT4 (pmol/l)	1.002	0.991–1.014	0.692	0.991	0.973–1.010	0.359
TSH (mIU/l)	0.000	0.000–55.746	0.093	0.000	0.000–436.441	0.115
TRAb (IU/l)	1.037	1.004–1.071	0.029	1.020	1.006–1.092	0.026
TPOAb (IU/ml)	1.000	0.999–1.001	0.450			
TGAb (IU/ml)	1.000	0.999–1.001	0.686			

Multivariate analyses are derived from univariate analysis adjusted for age, FT3, FT4, and TSH.

**Table 3:** Echocardiographic parameters of patients suffering from HHD complicated with PH (means ± SD).

Variables	Means ± SD
LVEF (%)	59.1 ± 7.8
E peak (cm/s)	113.8 ± 47.5
A peak (cm/s)	86.0 ± 33.9
E/A	1.37 ± 0.41
PASP (mmHg)	40.9 ± 9.5
LA (cm)	4.09 ± 0.60
RV (cm)	2.23 ± 0.30
LV (cm)	4.99 ± 0.59
RA (cm)	4.62 ± 0.91
*V*_max_ (cm/s)	292.8 ± 40.8
PG_max_ (mmHg)	34.9 ± 9.8

E, early ventricular filling velocity; A, late ventricular filling velocity; PASP, pulmonary arterial systolic pressure; LA, left atrial inner diameter; RV, right ventricular inner diameter; LV, left ventricular inner diameter; RA, right atrial inner diameter; *V*_max_, maximum velocity of blood flow in the artery of tricuspid valve; PG_max_, maximum pressure on both sides of the tricuspid valve.

**Table 4:** Correlation analyses of pulmonary arterial systolic pressure (PASP) with laboratory parameters.

Variables	Univariate analysis	
	*R* value	*P* value
FT3 (pmol/l)	0.020	0.868
FT4 (pmol/l)	0.155	0.194
TSH (mIU/l)	−0.082	0.491
TRAb (IU/l)	0.264	0.025
TPOAb (IU/ml)	0.121	0.312
TGAb (IU/ml)	0.085	0.480
LVEF (%)	−0.02	0.986
LA (cm)	0.075	0.533
RV (cm)	0.070	0.559
LV (cm)	−0.25	0.034
RA (cm)	0.064	0.593
*V*_max_ (cm/s)	0.744	≤0.001
PG_max_ (mmHg)	0.760	≤0.001

**Table 5:** Comparison of disease characteristics between groups with HHD complicated with PH (means ± SD).

Variables	*Q*1	*Q*2	*Q*3	Univariate analysis	
				*F* value	*P* value
*N*	24	23	25		
Age (years)	53.6 ± 12.9	44.5 ± 12.7	44.3 ± 12.9	4.096	0.21
BMI	22.2 ± 3.4	22.5 ± 2.3	22.1 ± 2.9	0.098	0.906
FT3 (pmol/l)	16.9 ± 7.9	22.6 ± 8.5	27.3 ± 13.7	5.889	0.004
FT4 (pmol/l)	55.5 ± 23.5	68.7 ± 24.7	81.5 ± 47.1	4.663	0.015
TSH (mIU/l)	0.0087 ± 0.014	0.0056 ± 0.0029	0.008 ± 0.008	0.653	0.523
TPOAb (IU/ml)	631.2 ± 568.4	1031.6 ± 438.7	899.5 ± 427.5	4.228	0.019
TGAb (IU/ml)	193.7 ± 194.7	314.2 ± 285.8	448.1 ± 792.8	1.542	0.221
PASP (mmHg)	37.2 ± 7.4	40.7 ± 9.8	44.6 ± 9.9	4.027	0.022
LVEF (%)	58.6 ± 8.6	57.6 ± 9.8	60.9 ± 3.9	1.113	0.335
LA (cm)	4.03 ± 0.62	4.31 ± 0.58	3.95 ± 0.58	2.403	0.098
RV (cm)	2.14 ± 0.24	2.33 ± 0.32	2.23 ± 0.31	2.656	0.77
LV (cm)	4.84 ± 0.43	5.10 ± 0.80	5.03 ± 0.51	1.277	0.285
RA (cm)	4.51 ± 0.75	4.77 ± 1.03	4.58 ± 0.96	0.498	0.610
*V*_max_ (cm/s)	274.6 ± 27.3	297.4 ± 42.3	305.9 ± 44.1	4.253	0.018
PG_max_ (mmHg)	30.5 ± 6.3	36.0 ± 10.5	38.2 ± 10.7	4.372	0.016

*Q*1: <11.596; *Q*2: 11.596–40.000; *Q*3: ≥40.000 IU/l; *Q*1, *Q*2, and *Q*3 were divided on the basis of the tertiles of the serum TRAb level.

## Data Availability

The data used to support the findings of this study are available from the corresponding author upon request.
